# Research trend of epigenetics and depression: adolescents' research needs to strengthen

**DOI:** 10.3389/fnins.2023.1289019

**Published:** 2024-01-05

**Authors:** Dongfeng Yuan, Yitong Meng, Zhongzhu Ai, Shiquan Zhou

**Affiliations:** ^1^Faculty of Pharmacy, Hubei University of Chinese Medicine, Wuhan, China; ^2^Hubei Shizhen Laboratory, Wuhan, China; ^3^Modern Engineering Research Center of Traditional Chinese Medicine and Ethnic Medicine of Hubei Province, Wuhan, China

**Keywords:** depression, epigenetics, adolescents, bibliometrics, research hotspots, VOSViewer

## Abstract

**Objective:**

With its high prevalence, depression's pathogenesis remains unclear. Recent attention has turned to the interplay between depression and epigenetic modifications. However, quantitative bibliometric analyses are lacking. This study aims to visually analyze depression epigenetics trends, utilizing bibliometric tools, while comprehensively reviewing its epigenetic mechanisms.

**Methods:**

Utilizing the Web of Science core dataset, we collected depression and epigenetics-related studies. Employing VOSViewer software, we visualized data on authors, countries, journals, and keywords. A ranking table highlighted field leaders.

**Results:**

Analysis encompassed 3,469 depression epigenetics studies published from January 2002 to June 2023. Key findings include: (1) Gradual publication growth, peaking in 2021; (2) The United States and its research institutions leading contributions; (3) Need for enhanced collaborations, spanning international and interdisciplinary efforts; (4) Keyword clustering revealed five main themes—early-life stress, microRNA, genetics, DNA methylation, and histone acetylation—highlighting research hotspots; (5) Limited focus on adolescent depression epigenetics, warranting increased attention.

**Conclusion:**

Taken together, this study revealed trends and hotspots in depression epigenetics research, underscoring global collaboration, interdisciplinary fusion, and multi-omics data's importance. It discussed in detail the potential of epigenetic mechanisms in depression diagnosis and treatment, advocating increased focus on adolescent research in this field. Insights aid researchers in shaping their investigative paths toward understanding depression's epigenetic mechanisms and antidepressant interventions.

## 1 Introduction

Depression is one of the most common mental disorders worldwide. It is characterized by a persistent low mood or lack of pleasure lasting for at least 2 weeks, accompanied by a constellation of cognitive or physiological abnormalities such as disrupted sleep, altered patterns of physical activity, and changes in eating habits. In severe cases, it can evoke feelings of pessimism, despair, distress, and even thoughts of death. Depression is considered a major public health concern, accounting for 40.5% of disability-adjusted life years (Whiteford et al., 2013; Januar et al., 2015). The World Health Organization ranks major depressive disorder (MDD) as the third leading cause of the global burden of disease and projects it to become the leading cause by 2030 (Patten et al., 2016; Malhi and Mann, 2018). The increasing prevalence of depression among the young is of particular concern. Over the past two decades, depression in adolescents has emerged as a prominent mental health issue (Paul and Usha, 2021), with a lifetime prevalence as high as 25%, compared to 5% in adults worldwide (Kessler et al., 2001; Herrman et al., 2022). Approximately half of all mental illnesses in adults have their onset during adolescence, and the risk of transitioning to depression is heightened. Alarmingly, up to 80% of adolescents affected by depression do not receive appropriate treatment (Kataoka et al., 2002; Jones, 2013), necessitating increased attention. The etiology of depression is complex, involving interactions among various factors such as psychosocial stress, genetics, and biological alterations. However, the precise mechanisms underlying its onset remain unclear (Mandelli and Serretti, 2013).

With the advancement of high-throughput sequencing techniques, an increasing body of evidence suggests that genetic factors play a significant role in the susceptibility to depression. To date, more than 100 genetic risk loci have been implicated, and the heritability of depressive disorders ranges between 30% and 50% (Bierut et al., 1999; Shadrina et al., 2018; Kendall et al., 2021; Wu et al., 2022). Recently, epigenetic modifications have gained prominence as crucial mechanisms contributing to the sustained elevation of depression risk. Epigenetics, first introduced by C. H. Waddington in 1942, refers to heritable and environment-modifiable changes in gene expression mediated through non-DNA coding mechanisms (Waddington, 1940; Sun et al., 2013). A notable correlation between depression and epigenetics exists (Park et al., 2019; Penner-Goeke and Binder, 2019). Epigenetic mechanisms, primarily involving DNA methylation, histone covalent modifications, microRNAs, chromatin remodeling, and gene imprinting, exert critical regulatory roles in processes such as growth, development, and metabolism (Ding et al., 2021; Park et al., 2021; Paoli et al., 2022). Epigenetic regulatory factors are involved in various pathways contributing to the pathogenesis of depression, including neurogenesis, synaptic plasticity, brain-derived neurotrophic factor expression, HPA axis regulation, neurotransmission, neuropeptide expression, neuroinflammation, and monoamine synthesis (Kocerha et al., 2015; Liu et al., 2020). Currently, research into epigenetics in the field of depression has garnered significant attention, emerging as a novel research hotspot. Understanding the progress, trends, and focal points in this domain is imperative for researchers engaged in relevant studies.

Bibliometric analysis is a scientific method used to assess published academic literature, identify data correlations, and predict the future development of specific research fields (Hicks et al., 2015). While some qualitative reviews have summarized the role of epigenetics in the onset and progression of depression, bibliometric methods have not yet been applied to this area of research. Qualitative reviews may carry potential subjective biases (Hosseini et al., 2018), emphasizing the necessity for a systematic evaluation of the relationship between epigenetics and depression using quantitative methodologies. Therefore, in order to achieve a comprehensive assessment, we focused on the most widely used and standardized database, Web of Science (WoS) (Zhu et al., 2023), and employed the VOSViewer software for quantitative analysis and visualization of literature (Ai et al., 2023).

In this study, through bibliometric analysis, we explored the interplay between depression and epigenetics over the past two decades, quantitatively analyzing and visualizing the literature in this field. This approach ensures results are quantifiable, more accurate, and objective, allowing for the elucidation of developmental trends in the field. We aimed to summarize the research focus and trends in the direction of epigenetics in depression. Furthermore, with a particular emphasis on advancements in the study of depression in adolescents, we discussed the current development status of epigenetic research in adolescent depression, aiming to provide researchers with a rapid overview of the field, guide future research directions, and provide clinical practice insights based on existing evidence.

## 2 Analytical methods

### 2.1 Data search strategy

In this study, we conducted a search within the WoS Core Collection for research related to epigenetics and depression. To establish a comprehensive literature repository concerning epigenetic research in depression, we performed searches from two perspectives. Firstly, considering the influence of factors like anxiety and other mental disorders on depression, the search strategy in WoS was formulated as follows: TS = (“depression” OR “anxiety” OR “depressive disorder^*^” OR “mental health disorder” OR “despondent” OR “gloomy” OR “depressive”). Secondly, to narrow the focus of the research to epigenetics, the search strategy in WoS was set as follows: TS = (“epigenetic” OR “epigenomic^*^” OR “DNA methylation” OR “methyltransferase^*^” OR “demethylase^*^” OR “Hypermethylation” OR “Hypomethylation” OR “histone modification” OR “histone methylation”OR “histone phosphorylation” OR “histone deacetylase^*^” OR “histone acetyltransferase^*^” OR “histone acetylization” OR “chromatin remodeling” OR “non-coding RNA^*^” OR “MicroRNA^*^” OR “micro RNA^*^”OR “micro-RNA^*^” OR “lncRNA^*^” OR “long ncRNA^*^” OR “long noncoding RNA^*^”OR “Long Non-Coding RNA^*^” OR “circRNA^*^” OR “Circular RNA^*^” OR “circ-RNA” OR “RNA modification” OR “miRNA” OR “N6-methyladenosine” OR “5-methylcytosine”).

### 2.2 Data screening strategy

**Figure 1:**
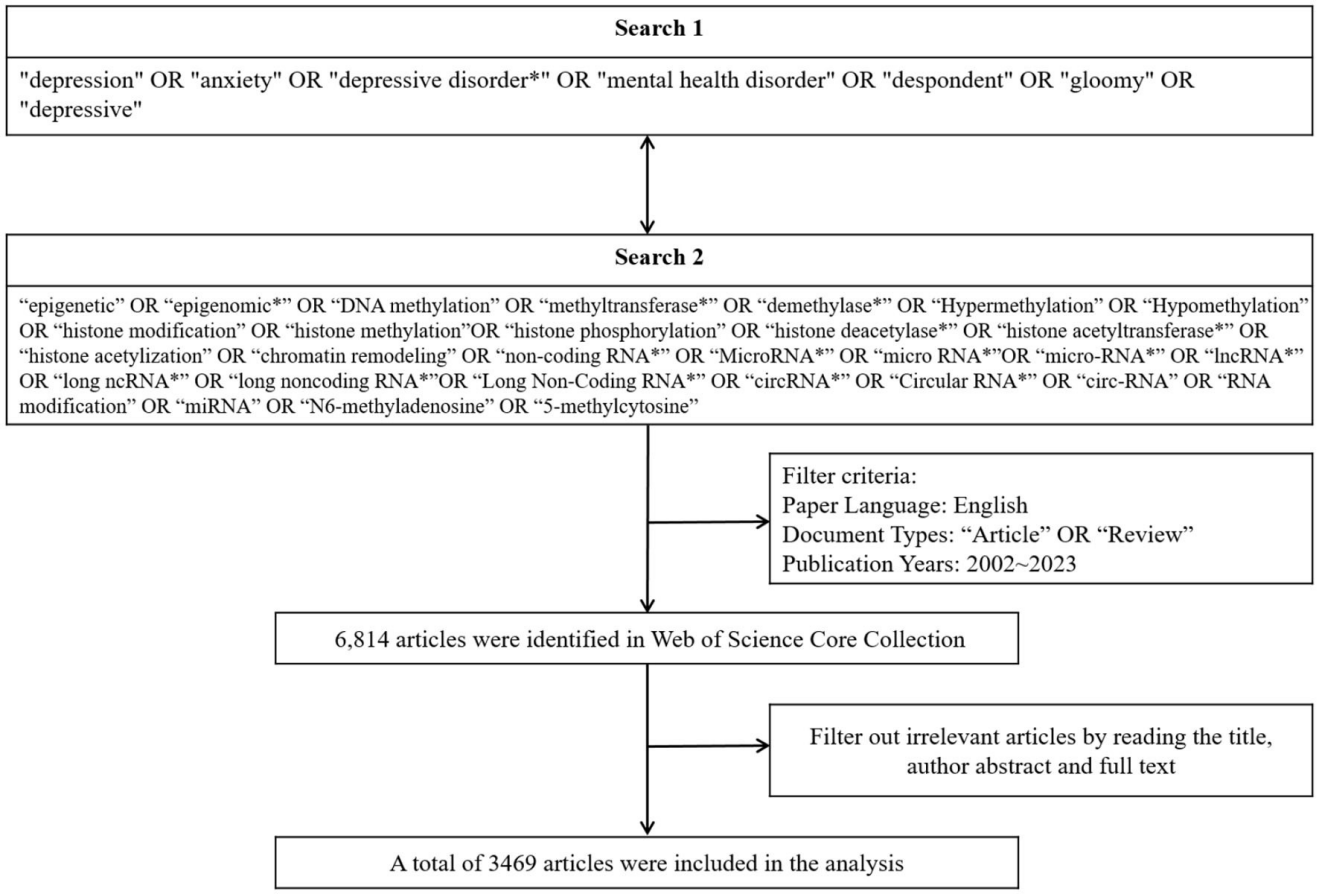
Data search strategy.

The screening procedures were as follows: The document types were limited to “Article” and “Review,” and the time was set from January 2002 to June 2023. Additionally, the language was restricted to English. A total of 6,814 articles were initially retrieved. Furthermore, throughout the entire process of screening and data collection, two independent reviewers identified potential studies based on screening criteria by reading article titles, abstracts, and full texts. Discrepancies were discussed, and consensus was reached. All data for this study were extracted by July 4, 2023. Following the screening process, a total of 3,469 articles were included. The process of data screening is illustrated in Figure 1.

### 2.3 Data analysis strategy

Descriptive statistics and bibliometric methods were employed to analyze the literature in the dataset, aiming to provide an overview of the research progress and status in the field of epigenetics in depression. The annual publication count and citation frequency were used to depict the developmental trends of this field over the past two decades. Ranking tables were compiled based on publication output from countries, institutions, authors, and journals to identify the key leaders in the field of epigenetics in depression.

VOSviewer, a renowned literature analysis software, utilizes relationships between terms in academic literature (such as authors, journals, and keywords) to visually represent scientific research trends within a certain domain. VOSviewer's analysis units encompass countries, authors, journals, keywords, and more, depending on the focus of the analysis and the type of database. In the visualization, nodes represent the analysis units, and their size reflects their importance in the visualization. Connections between nodes indicate relationships, with thicker lines indicating stronger connections. Nodes of the same color are clustered together, representing related units within the same cluster (Van Eck et al., 2010).

In this study, we first conducted a collaboration analysis of countries and authors to explore the collaborative patterns in the field of epigenetics in depression. Subsequently, we utilized journal co-citation analysis to unveil the potential disciplinary foundations of this field. Finally, through the visualization analysis of keywords, we delved into the research hotspots and developmental trends in the field.

## 3 Results

### 3.1 Development trend of epigenetic research on depression

**Figure 2:**
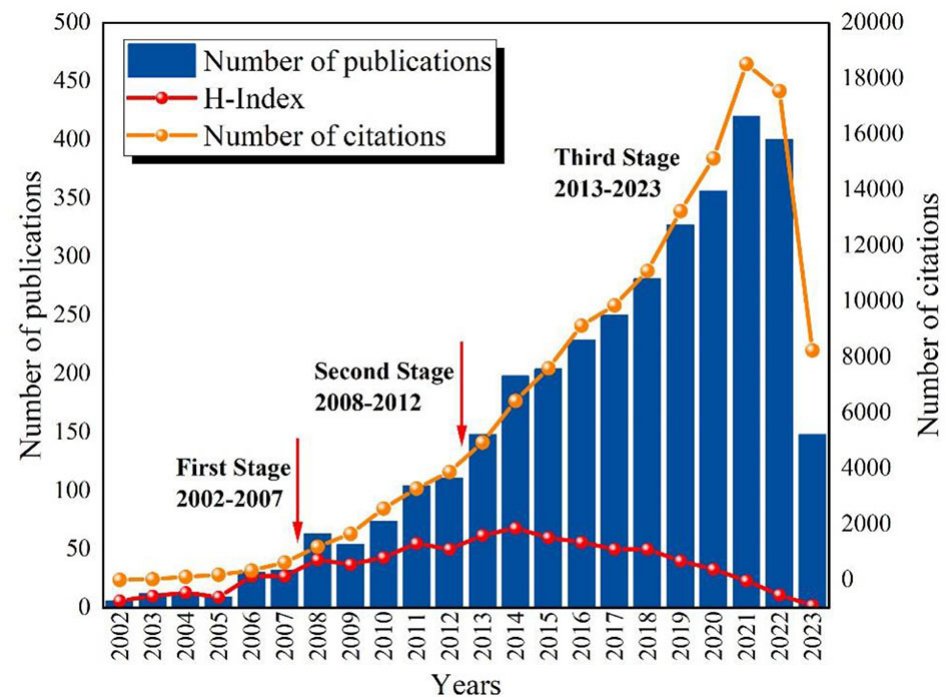
The annual number of publications, citations and H-Index on epigenetics and depression from 2002 to 2023.

As shown in Figure 2, the number of publications and total citations related to epigenetics in depression exhibited an overall upward trend from 2002 to 2023. This indicates a sustained interest among scholars in the research of epigenetics in depression. Broadly speaking, the research in this field can be categorized into three stages: Initial Stage (2002–2007): During this period, the related research experienced slow growth, with the annual publication count remaining below 50. This suggests that attention and interest in the topic were relatively low. Development Stage (2008–2012): The number of publications and citation frequencies increased during this phase and maintained a steady pace. Take-off Stage (2013–2023): Over the course of these 10 years, both the number of publications and citation frequencies significantly increased, exhibiting a higher growth rate. The peak was reached in 2021 (publications: 420, citations: 18524). Although there was a slight decrease in the number of publications in 2022, it remained at a relatively high level. Furthermore, the trend in the data indicate that the research interest in the relationship between depression and epigenetic modifications is continually increasing. Scholars have begun to closely investigate the impact of depression on epigenetic modifications, as evidenced by the sustained rise in publications and citations.

### 3.2 Predominant leader in epigenetic research on depression

#### 3.2.1 Leading publication source

In total, 959 journals have published articles from the included 3,469 articles in the dataset. Table 1 presents the top 10 core journals in terms of publication volume in this field. These journals collectively published 672 papers, accounting for 19.37% of the total papers. In 2022, the impact factors (IF) of these top 10 journals ranged from 3.7 to 11. *Translational Psychiatry* made the most significant contribution, accounting for ~3% of the publications in the dataset (n = 108). *Journal of Affective Disorders* ranked second with 89 publications. Notably, despite being an interdisciplinary and relatively young journal, *Plos One* also published a substantial amount of research in this field and secured the third position. Additionally, several journals related to molecular science have played crucial roles in the development of this field, such as *International Journal of Molecular Sciences* (57), *Molecular Neurobiology* (54), and *Molecular Psychiatry* (54), ranking fourth, seventh, and eighth, respectively. Among these top 10 journals, 50% are in Q1, and the rest are in Q2, indicating the reliability and high quality of these studies. According to citation frequency, *Biological Psychiatry* appears to be one of the most popular and recognized journals in this field.

**Table 1:** Top 10 publication sources in terms of publications.

**Name**	**Number**	**Citations**	**Average citation**	**IF (2022)**	**JCR(2022)**	**H-index**
Translational Psychiatry	108	4,846	44.87	6.8	Q1	41
Journal of Affective Disorders	89	2,045	27.02	6.6	Q1	27
Plos ONE	84	4,500	53.57	3.7	Q2	40
International Journal of Molecular Sciences	72	715	9.93	5.6	Q1	16
Journal of Psychiatric Research	57	2,017	35.39	4.8	Q2	22
Frontiers In Psychiatry	55	963	17.51	4.7	Q2	18
Molecular Neurobiology	54	933	17.28	5.1	Q2	19
Molecular Psychiatry	54	3,645	67.5	11	Q1	31
Scientific Reports	52	986	18.96	4.6	Q2	19
Biological Psychiatry	47	5,129	109.13	10.6	Q1	33

#### 3.2.2 Leading authors, institutions, and countries/territories

Among the total of 3,469 articles, 82 major countries/regions have contributed to research in the field of epigenetic modifications in depression. Table 2 presents the top 10 countries/regions, institution and authors by publication count. The top five countries or regions by publication count are the United States, China, Germany, Canada, and the United Kingdom. The United States ranks first with 1,203 articles (34.67%), followed by China with 744 articles (21.44%), Germany with 332 articles (9.57%), Canada with 297 articles (8.56%), and the United Kingdom with 234 articles (6.74%). The remaining countries listed in the table are mainly distributed across Europe (Italy, Netherlands, France, etc.), East Asia (Japan), and Oceania (Australia). Overall, research on the relationship between depression and epigenetics has received varying degrees of attention worldwide, particularly gaining more focus in developed countries. Considering the average citation count per paper, France (57.61) ranks first, followed by Canada (56.8) and the United States (55.27). It's worth noting that China's average citation frequency in this field (16.93) is significantly lower than other countries, indicating a potential lower quality of research. Therefore, there is a need to increase research investment, improve research quality, and enhance international influence and competitiveness in this field.

**Table 2:** Top 10 countries/regions, institutions, and authors in terms of publications.

	**Rank**	**Name**	**Publications**	**Citations**	**Average citation**	**H-index**
Country	1	USA	1,203	66,493	55.27	126
	2	China	744	12,597	16.93	54
	3	Germany	332	16,861	50.79	67
	4	Canada	297	16,869	56.8	64
	5	England	234	11,196	47.85	60
	6	Italy	212	8,494	40.07	48
	7	Australia	162	8,034	49.59	50
	8	Netherlands	140	6,272	44.8	45
	9	Japan	135	5,742	42.53	42
	10	France	109	6,280	57.61	41
Institution	1	Mcgill University	121	7,932	65.55	50
	2	Harvard University	116	9,755	84.09	45
	3	University of London	110	5,846	53.15	45
	4	Icahn School of Medicine At Mount Sinai	105	10,380	98.86	50
	5	University of California System	101	5,787	57.3	36
	6	USA Department of Veterans Affairs	98	5,858	59.78	42
	7	Veterans Health Administration (VHA)	98	5,858	59.78	42
	8	University of Texas System	88	6,953	79.01	35
	9	King's College London	87	5,036	57.89	39
	10	Emory University	86	5,117	59.5	37
Author	1	Gustavo Turecki	58	3,413	58.84	32
	2	Nestler Eric J	51	9,729	190.76	35
	3	Domschke Katharina	41	1,437	35.05	23
	4	Zhang Yi	38	997	26.24	18
	5	Binder Elisabeth B	33	3,601	109.12	26
	6	Dwivedi Yogesh	33	1,822	55.21	22
	7	Wang Yu	27	864	32	12
	8	Pandey Subhash C	26	926	35.62	17
	9	Deckert Juergen	26	1,522	58.54	21
	10	Li Ying	25	339	13.56	9

The top 10 research institutions by publication count in the dataset for studies on depression and epigenetics are shown in Table 2. McGill University (121) ranks first, followed closely by Harvard University (116). Additionally, contributions from UK institutions like University College London (110) and King's College London (87) have also been significant in this field. Among the top 10 institutions, seven are from the United States, two from the United Kingdom, and one from Canada, which might explain the substantial lead of the United States in terms of publication output compared to other countries.

The top 10 authors collectively contributed 358 papers, accounting for 10.32% of the total research output in this field. Professor Gustavo Turecki holds the distinction of being the most prolific researcher in this area, with 58 related papers, securing the top position. Professor Nestler Eric J and Professor Domschke Katharina closely follow with 51 and 41 related papers, respectively. Moreover, Professor Nestler Eric J boasts an average citation frequency of 190.76, significantly higher than other authors, indicating his substantial influence and impact in this field.

### 3.3 Exploring scientific collaboration network for epigenetic research on depression

**Figure 3:**
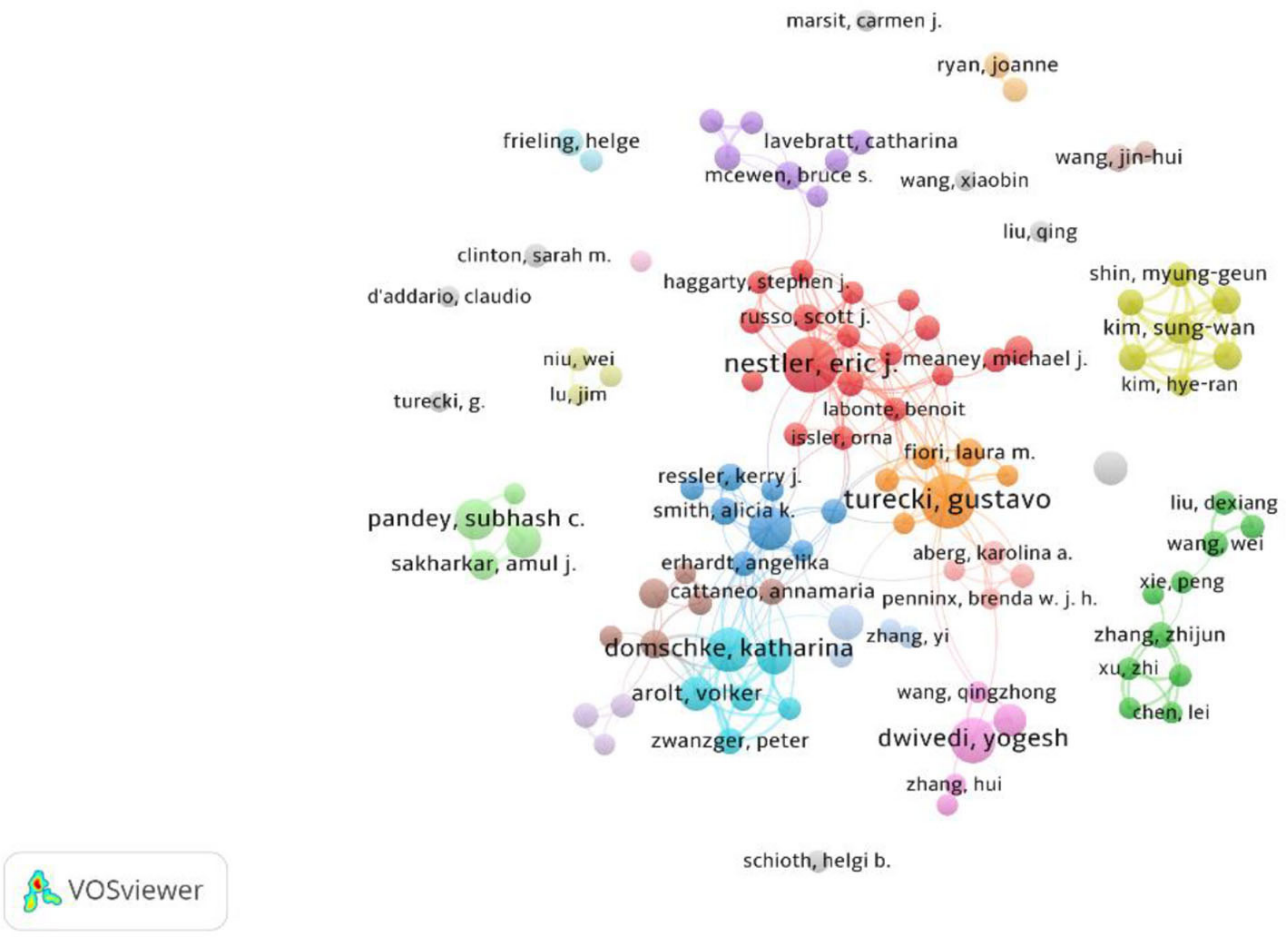
Scientific collaboration networks between authors.

In this study, we employed VOSViewer to explore the scientific collaboration patterns in the field of epigenetics in depression over the past 20 years, including collaborations among researchers and countries/regions. The collaboration network among researchers is illustrated in Figure 3. In this analysis, only researchers who have published at least eight papers within the dataset were included, resulting in 106 nodes and 251 connections. Clearly, the field comprises multiple collaborative clusters, with 10 multinational collaboration networks situated at the center of the graph, indicating close connections and numerous researchers. For instance, the largest collaboration cluster (Red cluster) is composed of 18 researchers from the United States, Canada, the Netherlands, and China. The collaboration group represented by Binder Elisabeth B (Blue cluster) consists of eight researchers from the United States, Germany, and Israel. The collaboration group represented by Turecki Gustavo (Orange cluster) includes six researchers from Canada, Germany, and France. These three groups are centrally positioned in the graph and are significantly connected to other collaboration clusters (such as Purple cluster, Cyan cluster, Brown cluster), signifying their pivotal role and outstanding contributions to the field's development. Furthermore, some regional collaboration clusters also merit attention. For example, the collaboration group represented by Zhang Zhijun (Green cluster) in the lower right portion of the graph comprises 10 researchers from regions including Nanjing, Jinan, and Chongqing in China. The collaboration group represented by Kim Sung-Wan (Yellow cluster) in the upper right part of the graph includes seven researchers from Gwangju, Korea. The collaboration group represented by Subhash C Pandey (Light green cluster) in the lower left part of the graph comprises four researchers from the University of Illinois at Chicago in the United States. These groups also play essential roles in epigenetics research on depression, but their collaborations tend to be more localized, with limited interactions with other research institutions, regions, or countries. Meanwhile, smaller academic teams are distributed around the periphery of the network graph, suggesting their relatively lesser influence.

**Figure 4:**
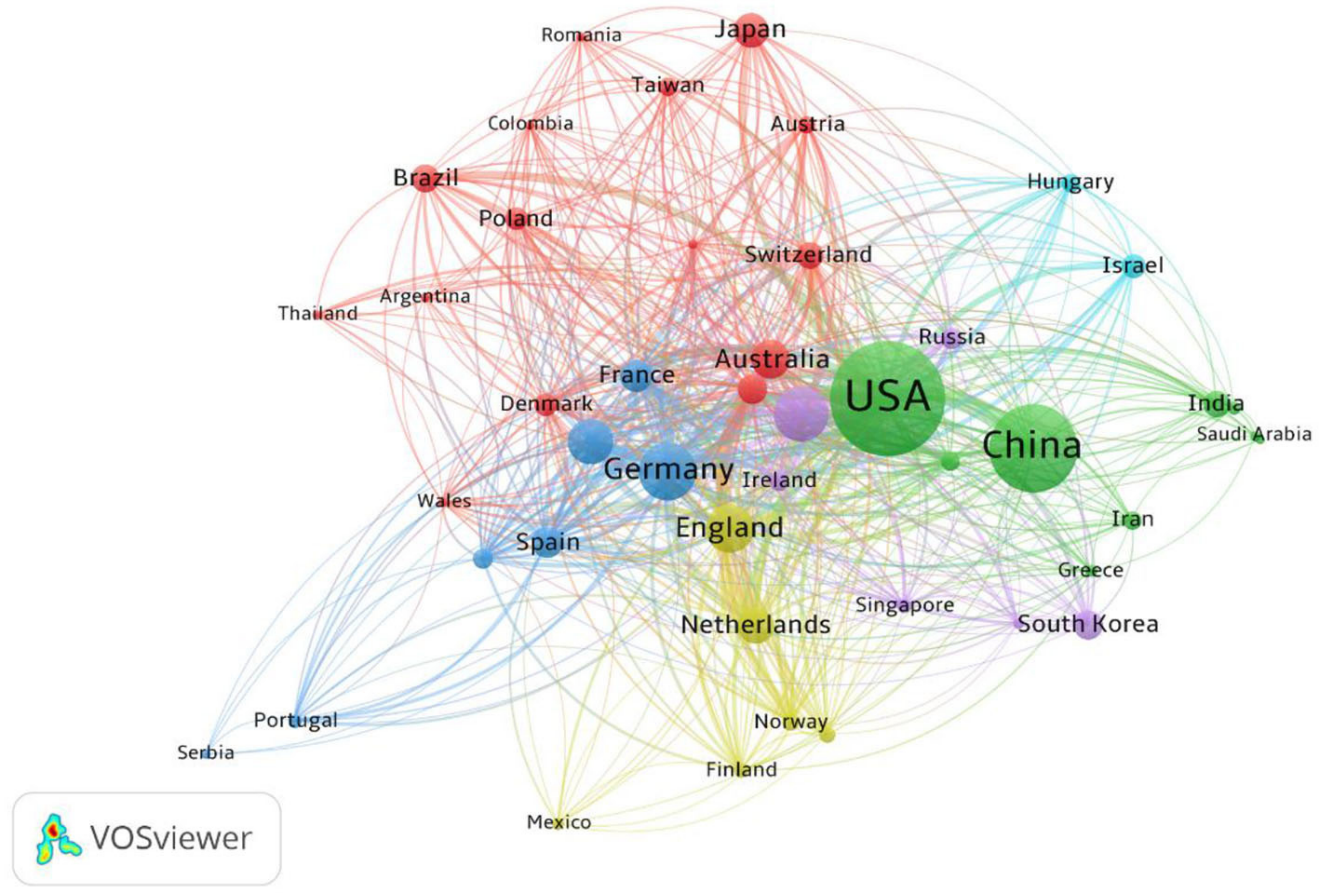
Scientific collaboration networks between countries/territories.

The international collaboration network in the field of depression epigenetics research is illustrated in Figure 4. In this analysis, only countries that have published at least 10 papers within the dataset were included, resulting in 45 nodes and 602 connections. The United States, represented by the largest node at the center of the network graph, forms strong collaborative relationships (Green cluster) with other countries or regions such as China, India, Greece, etc., highlighting its prominent influence in the field. Overall, international collaboration in this field is significantly influenced by language, geography, and cultural backgrounds. For example, the largest international collaboration cluster (Red cluster) consists of 11 European countries, two South American countries, and two Asian countries. They share similar cultural backgrounds, with English being their primary language. Additionally, a multinational collaboration cluster (Blue cluster) led by Germany consists of seven European countries, geographically closer to each other. Unfortunately, no influential Asian multinational collaboration cluster was identified in depression epigenetics research. This suggests that the research in this field is predominantly led by the United States and some European countries. However, despite this, the issue of depression in Asian populations should not be overlooked. Asian countries need to strengthen their collaboration efforts, paying more attention to depression patients of Asian descent and jointly exploring the epigenetic mechanisms involved (Song et al., 2011; Yu et al., 2016).

### 3.4 Revealing the disciplines underlying the foundations of epigenetic research on depression

**Figure 5:**
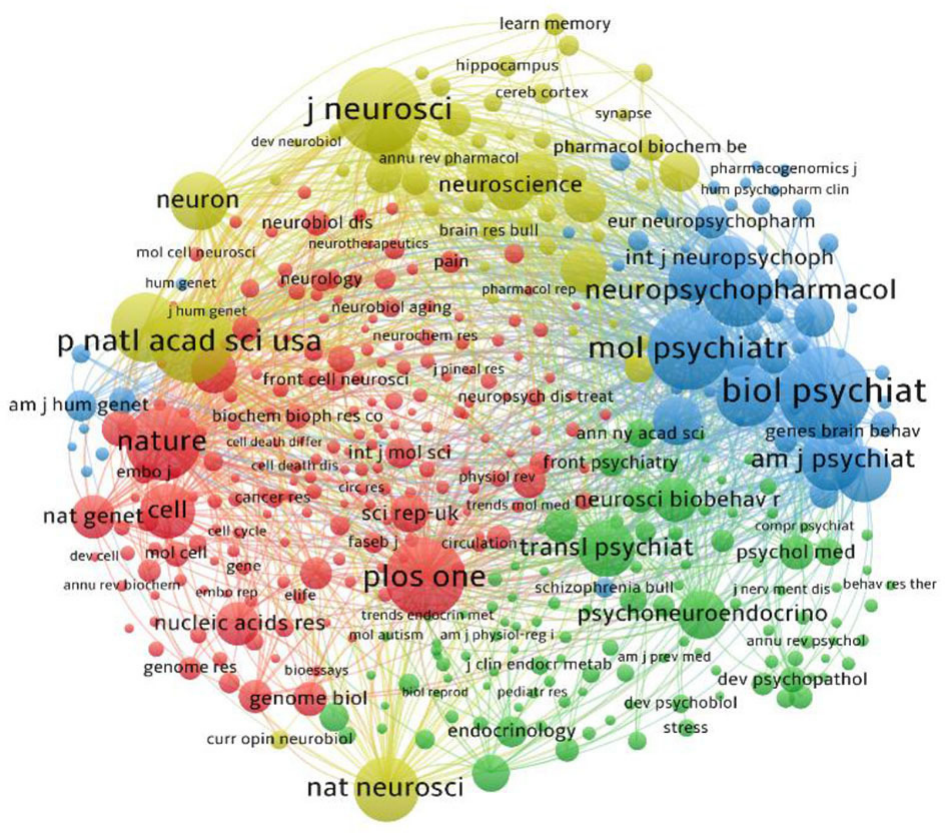
Map of clustered network journals based on co-citation data.

To explore the potential disciplinary foundations of depression epigenetics research, we conducted a journal co-citation analysis on the literature within the dataset using VOSViewer. Co-citation refers to the situation where two journals are both cited by a third journal. The more frequently a pair of journals are co-cited, the stronger their co-citation relationship, indicating a strong theoretical and semantic basis (Hernández-Torrano et al., 2020). In this analysis, we considered only journals that were co-cited at least 100 times, resulting in 419 nodes and 86,001 connections, as shown in Figure 5. Overall, the results of this study suggest that depression epigenetics research is interdisciplinary to some extent, primarily originating from the fusion of neuroscience and molecular medicine. Specifically, the network graph indicates that depression epigenetics research is constructed by integrating knowledge from four interconnected disciplines. In the upper part of the network graph, the Yellow cluster predominantly integrates journals from the fields of neuroscience and brain science. In the lower right part of the graph, the Green cluster brings together journals from psychiatry, endocrinology, and behavioral biology. On the left side of the graph, the Red cluster represents contributions from molecular science, genetics, and some comprehensive journals in the field. In the upper right part of the graph, the Blue cluster encompasses journals related to neuropharmacology, molecular psychiatry, and other psychiatry-related areas.

### 3.5 Finding the hotspots and frontiers in epigenetic research on depression

**Figure 6:**
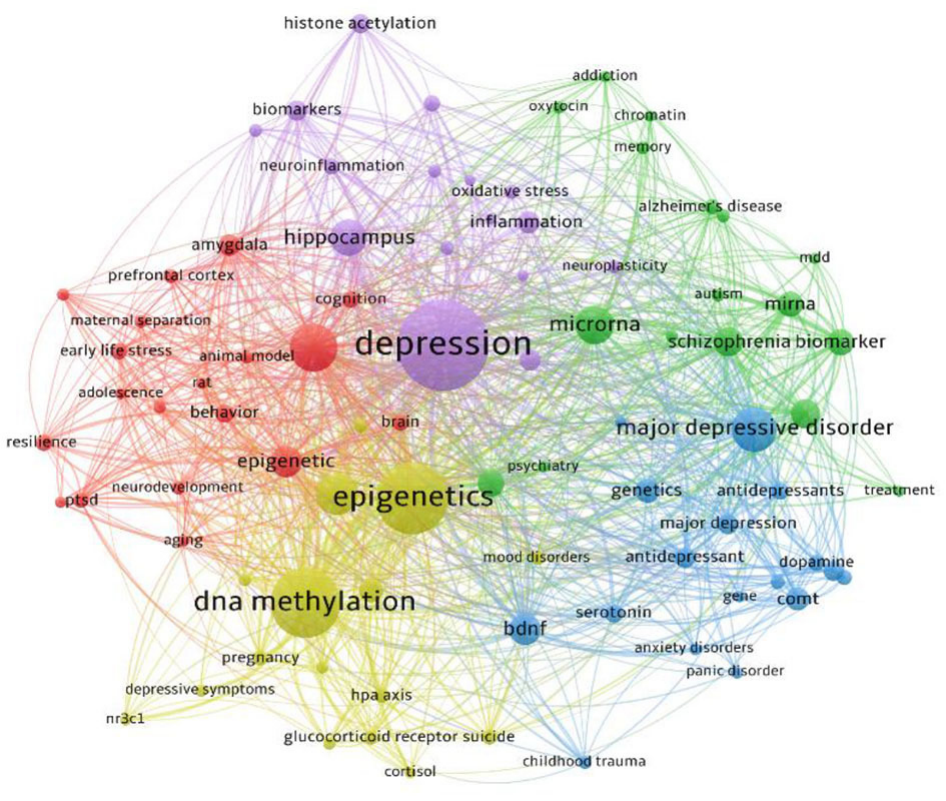
Co-occurrence network map of keywords.

The keyword co-occurrence map provides insights into the research hotspots within the field of epigenetics in depression. Keyword co-occurrence refers to the frequency of appearance of two or more keywords in the same article, illustrating their relationships. The research hotspots in the field of epigenetics in depression from 2002 to 2023 are depicted in Figure 6. In this analysis, only keywords that appeared at least 20 times were included, resulting in the generation of 85 nodes and 1,554 edges. The top 10 keywords in terms of frequency are presented in Table 3.

**Table 3:** Top 10 high-frequency keywords.

**Rank**	**Keywords**	**Occurrences**	**Total link strength**
1	Depression	661	1,235
2	Epigenetics	451	982
3	DNA methylation	429	856
4	Anxiety	228	495
5	Major depressive disorder	201	346
6	Stress	181	457
7	Micro RNA	160	286
8	Hippocampus	144	237
9	BDNF	114	235
10	Schizophrenia	103	242

Keywords represent the core themes of articles, and higher frequencies indicate greater research interest in a particular topic. Among the dataset, the five highest-frequency keywords are “depression” (661 occurrences), “epigenetics” (451 occurrences), “DNA methylation” (429 occurrences), “anxiety” (228 occurrences), and “major depression disorder” (201 occurrences). This indicates that depression, anxiety, and major depressive disorder have become central focus points for researchers globally. Notably, “DNA methylation” ranks third with 429 occurrences, suggesting that this topic has been extensively explored in the past two decades and holds significant research potential. Additionally, the presence of “MicroRNA” (160 occurrences) and “BDNF” (114 occurrences) signifies considerable interest among researchers in the context of epigenetic studies related to depression. This trend also underscores the rise of epigenetics as a promising avenue for enhancing our understanding of depression, diagnostics, and therapeutic interventions, aligning with the findings of Hack et al.'s (2019) study.

The keyword clustering results (Figure 6) reveal that five major themes encompass the research hotspots in epigenetics and depression over the past two decades. First, the impact of early-life stress on epigenetic modifications and depression development has garnered extensive attention, as evidenced by the high-frequency keywords such as “early life stress,” “maternal separation,” “adolescence,” and “ptsd” (red cluster). Second, the influence of microRNAs on various psychiatric disorders constitutes another extensively studied topic, with keywords such as “microrna,” “alzheimer's disease,” “mdd,” “autism,” and “treatment” (green cluster). Third, the genetics and gene research related to depression and antidepressants has emerged as a prominent hotspot, encompassing keywords like “major depression,” “genetics,” “antidepressants,” “gene,” “bdnf,” and “dopamine” (blue cluster). Fourth, the impact of DNA methylation on depression is also a significant focus, represented by keywords like “dna methylation,” “depressive symptoms,” “nr3c1,” and “hpa axis” (yellow cluster). Lastly, the effect of histone acetylation on depression, including keywords such as “depression”, “histone acetylation”, “hippocampus”, “neuroinflammation” constitutes another critical theme (purple cluster). Importantly, these five clusters interweave, indicating the interconnectedness of these research topics.

**Figure 7:**
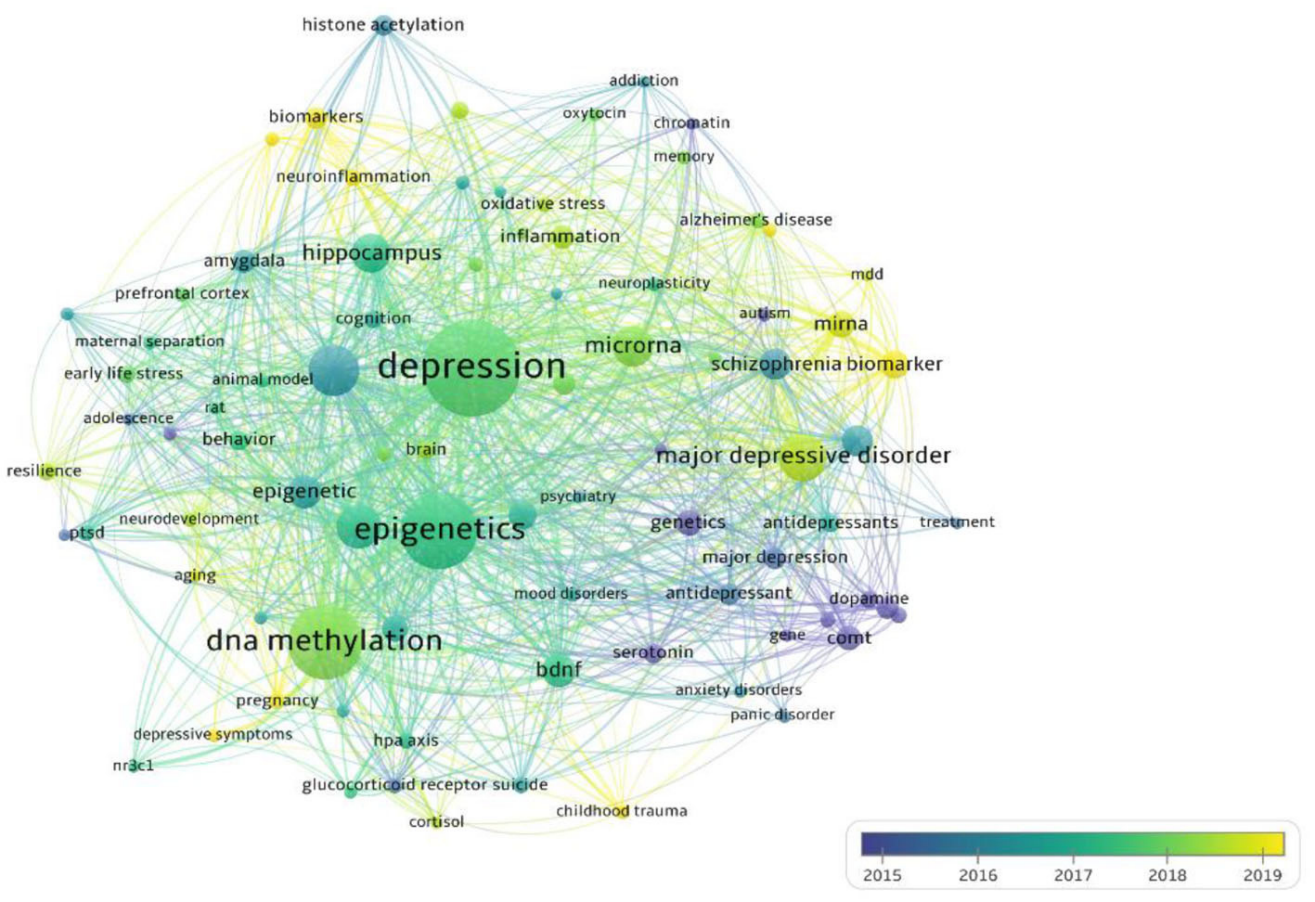
Average year map of keywords used from 2002 to 2023.

In order to investigate the changing trends of research hotspots in this field from 2002 to 2023, we conducted a time-based visual analysis of keywords, as depicted in Figure 7. The shades of blue denote keywords that had an earlier average appearance year, while yellow shades indicate keywords with a later average appearance year. Node size corresponds to the level of research interest, and the legend provides the average year information. It is evident that the initial research hotspots in this domain were predominantly centered around aspects of genetics, genes, neurotransmitters, and depression. Around 2017, the research focus transitioned toward the nexus of depression, epigenetics, and DNA methylation. By around 2019, keywords like “miRNA,” “biomarker,” “neuroinflammation,” and “childhood trauma” have emerged as recent research hotspots.

## 4 Discussion

### 4.1 General status and trends in epigenetics and depression

Based on bibliometric methodology, during the course of our research, we initiated the retrieval process within four major databases: WoS, PubMed, Scopus, and Google Scholar. It was observed that WoS contained the largest volume of literature, and after the elimination of duplicates through screening, WoS remained the predominant source of primary literature. Moreover, WoS exhibited superior literature quality, particularly in terms of citation accuracy, compared to other databases (Falagas et al., 2008; Wang and Waltman, 2016; Zhang et al., 2023). Consequently, for this study, metadata extraction was chosen from the WoS database to facilitate data visualization, aiming to provide a more intuitive and lucid understanding of the present status, focal points, and cutting-edge aspects within this field. Overall, over the past two decades, the volume of publications pertaining to epigenetics in depression has steadily grown, reaching a zenith in 2021, signifying the elevated research interest in this domain. The journal with the highest publication count was *Translational Psychiatry*, while *Biological Psychiatry* attained the highest average citation frequency. The United States and its affiliated research institutions made substantial contributions to the augmentation of publication output in this field. Notably, McGill University from Canada emerged as the most prolific institution in terms of publication count. Preeminent authors with the most extensive publication records and influential contributions were Professor Gustavo Turecki and Professor Nestler Eric J. The outcome of keyword analysis disclosed that the research hotspots in this field predominantly centered around genetics, gene modifications, and neurotransmitters related to depression. Furthermore, the emergence of frequently occurring keywords in recent years, such as “miRNA,” “biomarker,” “neuroinflammation,” and “childhood trauma,” delineated the latest research trends and forefront areas within this field.

Collaboration within this field needs to be strengthened, encompassing both international and interdisciplinary cooperation. The results of collaboration network analysis indicate that collaborations among authors in this field are primarily concentrated in developed countries such as the United States, Canada, Germany, and France. Collaborations among authors from developing countries tend to be limited to domestic partnerships, with even fewer contributions from authors in underdeveloped regions. Collaborative efforts between countries are often influenced by factors such as language, cultural background, and geographical location, displaying a preference for localized collaborations. David et al. emphasized that multinational collaborative research has become a means to enhance research quality, optimize resource utilization, and expand influence (Hsiehchen et al., 2015), and the disparities in research capabilities between developed and developing nations, along with their implications for global health research trends, can no longer be disregarded (Titilola, 2011). Journal co-citation analysis reveals that research in this field is, to a certain extent, interdisciplinary. A multitude of journals from various disciplines collectively form the disciplinary framework of epigenetic research in depression. This framework predominantly revolves around neuroscience and molecular science, supplemented by behavioral science, endocrinology, neuropharmacology, and other health-related disciplines. Robust interdisciplinary partnerships can integrate multi-level “etiologies” of behavioral socioecological models with the molecular, cellular, and ultimate physiological foundations of health and disease. This integration holds the potential for breakthroughs that will improve public health (Mabry et al., 2008). Therefore, we hope that, developed countries will engage in further collaborations with nations from underdeveloped regions within this field. We also encourage researchers from different disciplinary to establish robust collaborative relationships, resulting in higher-quality research outcomes and greater applicability of relevant studies.

### 4.2 Epigenetic modification in depression

#### 4.2.1 Genetics and gene research on depression

Depressive disorder exhibits significant heritability, a fact supported by the third largest cluster of keywords (Figure 6). A meta-analysis based on five family studies indicated a heritability range of 31% to 42% for depression (Sullivan et al., 2000). However, despite extensive research including large-scale genome-wide association studies (GWAS) and whole genome sequencing (WGS), and significant achievements in the genetics of psychiatric disorders such as schizophrenia (Schizophrenia Working Group of the Psychiatric Genomics Consortium, 2014) and bipolar disorder (Ikeda et al., 2018), progress in identifying genetic variants related to MDD significantly lags behind. For instance, Lewis et al. conducted a GWAS on 1,636 positive patients and 1,594 negative controls in the UK, revealing suggestive evidence of an association between MDD patients and SNPs in BICC1 (Lewis et al., 2010). Additionally, a meta-analysis with a larger sample size (5,763 cases and 6,901 controls) failed to detect genome-wide significant single nucleotide polymorphisms (SNPs) (Wray et al., 2012). This phenomenon might be attributed to inadequate sample size and considerable heterogeneity, thus necessitating subsequent large-scale studies and efforts to reduce heterogeneity. This assertion is supported by a study by Wray et al. (2018), who performed a genome-wide association meta-analysis on 135,458 cases and 344,901 controls, identifying 44 independent and significant loci.

Numerous studies indicate the involvement of specific genes in the pathophysiology of depression. The most frequently studied genes include brain-derived neurotrophic factor (BDNF), serotonin receptors (5-HT), serotonin transporter protein (SLC6A4 or 5-HTT), and catechol-O-methyltransferase (COMT) (Mehta et al., 2010; Seripa et al., 2013). Changes in the expression levels of these genes have been linked to resilience, depression, and anxiety (Lacerda-Pinheiro et al., 2014). Additionally, the role of inflammatory processes in the pathogenesis of depression has garnered attention in recent years. For instance, Al-Hakeim et al. (2015) found elevated serum levels of IL-6, IL-18, TNF-α, and sIL-2R in MDD patients compared to controls, while Draganov et al. (2019) suggested that polymorphisms in the IL1-β and IL6R genes might influence treatment response in severe depressive disorder. Genome-wide sequencing results have also revealed correlations between variants associated with inflammatory responses and depression (Wong et al., 2017). These findings suggest that with the advancement of GWAS and gene sequencing technologies, new candidate genes will continue to emerge, aiding further exploration of the biological mechanisms underlying depression and the development of antidepressant medications.

#### 4.2.2 DNA methylation

The keyword “DNA methylation” appears 429 times and is situated within the Yellow cluster (Figure 6), representing one of the most extensively studied hotspots in the realm of epigenetic modifications. This underscores that the majority of investigations exploring the link between depression and epigenetic changes are focused on DNA methylation. Notably, within this clustering, “HPA axis” captures our attention. The hypothalamic-pituitary-adrenal (HPA) axis, comprising the hypothalamus, pituitary gland, and adrenal cortex, forms a complex system with crucial regulatory functions within the body. Extensive research indicates a connection between stress exposure, HPA axis dysregulation, and susceptibility to neuropsychiatric disorders. Such connections exist across various developmental stages. Exposure to stress in different forms, durations, and intensities leads to maladaptive medium-to-long-term HPA axis responses in the brain. Changes in glucocorticoid signaling and alterations in epigenetic modifications are pivotal mechanisms that impact the HPA axis and its associated cascades (Lee and Sawa, 2014; Bockmühl et al., 2015).

Early-life stress (ELS) exerts negative effects on cognitive and emotional functions, and these effects are mediated through dysregulation of the HPA axis, abnormal neurotrophic factor levels, and their impacts on hippocampal function. Brain-derived neurotrophic factor (BDNF) has been implicated in the pathophysiological response to depression. Studies have indicated that animals exposed to repeated stress exhibit significant cognitive impairments, marked elevation of plasma corticosterone, a significant reduction in hippocampal BDNF levels, and a significant decrease in overall DNA methylation of the stress-exposed animals' hippocampal genome (Makhathini et al., 2017). Maternal separation (MS) in rats induced epigenetic modifications of the BDNF exon I promoter, and these changes were prevented by antidepressant treatment in adulthood (Park et al., 2018). A follow-up study of 85 Chinese depressed patients undergoing Escitalopram treatment revealed that after 8 weeks of treatment, the average methylation level of BDNF was significantly lower than that at the end of the treatment (Wang et al., 2018). Furthermore, increased DNA methylation levels of the BDNF gene have been linked to depression in the general population (Fuchikami et al., 2011). These findings suggest that epigenetic changes of BDNF may play a crucial role in the pathophysiological progression and therapeutic implications of depression. Maternal prenatal psychosocial stress is associated with dysfunction of the infant HPA axis, with DNA methylation alterations considered a potential underlying mechanism. Studies have confirmed that maternal distress and anxiety lead to slight increases in methylation at specific CpG sites of NR3C1 in newborns (Mansell et al., 2016). In another longitudinal study involving 167 children aged 6–9, prenatal depression was associated with lower cortisol release in boys and higher cortisol release in girls (Stonawski et al., 2019). Additionally, prenatal depressive symptoms were linked to methylation changes in the glucocorticoid receptor gene (NR3C1), mineralocorticoid receptor gene (NR3C2), and serotonin receptor gene (SLC6A4), exhibiting some gender-specific effects [*p* = 0.012–0.040, (2)(*p*) = 0.03–004], with an indirect effect = 0.07, 95% confidence interval [−0.16, −0.02]. Wiley et al. discovered that worsening maternal late-pregnancy anxiety was associated with lower methylation levels of the infant OXTR2CpG2, offering the first evidence that oxytocin receptor methylation might contribute to regulation of infant HPAA during the neonatal period (Wiley et al., 2023). Animal experiments have also demonstrated that the methylation status of the NR3C1 gene in offspring is sensitive to prenatal stress, prenatal stress is associated with demethylation of the corticotropin-releasing hormone (CRH) promoter, leading to anxiety-like behavior during adolescence and heightened reactivity of the HPA axis (Xu et al., 2014; Öztürk et al., 2022). In conclusion, these findings suggest that maternal prenatal stress and early-life stress may disrupt normal HPA function through epigenetic mechanisms.

Furthermore, the function of the HPA axis is mediated by the glucocorticoid receptor (GR), influenced by the epigenetic mechanism of DNA methylation (Tyrka et al., 2016). A clinical study involving 80 hospitalized depressive patients and 58 healthy controls revealed that depressed patients exhibited reduced cortisol responsiveness to the Trier Social Stress Test (TSST), along with increased methylation levels of SLC6A4 and NR3C1 in serum (Bakusic et al., 2020). Similarly, in a larger study involving 349 volunteers, depressed volunteers displayed higher levels of NR3C1 DNA methylation and lower cortisol levels (Borçoi et al., 2020), providing evidence for the role of glucocorticoid level changes, NR3C1, and SLC6A4 DNA methylation in HPA axis dysregulation in depression patients, warranting further exploration. Methylation of exon 1 (F) of the glucocorticoid receptor gene NR3C1 is associated with early stress exposure and risk of developing psychiatric disorders. Studies have indicated an association between methylation of NR3C1 exon 1F, particularly CpG2 methylation, and depression (Kang et al., 2018). Palma-Gudiel et al. assessed 48 pairs of monozygotic twins (*n* = 96 subjects) with a history of lifetime anxiety and depressive disorders, and DNA extracted from peripheral blood was analyzed for epigenetic changes, revealing that NR3C1 promoter exon 1_D_ becomes a suggestive novel target for stress-related disorders sensitive to early adversity-related epigenetic changes (Palma-Gudiel et al., 2018). DNA methylation levels in the promoter region of NR3C1 were associated with childhood maltreatment (CM) and depression, suggesting that CM exposure may biologically embed these critical HPA axis genes (Bustamante et al., 2016). FKBP5 is involved in regulating glucocorticoid receptor (GR) sensitivity, and GR resistance and associated stress hormone dysregulation are among the strongest biological findings in severe depression. The extent of this resistance may be modulated by FKBP5 polymorphism, with studies showing that only depressed patients carrying the risk allele FKBP5rs1360780 exhibit significant GR resistance (Menke et al., 2013). Additionally, FKBP5 thought to link maltreatment to mental and physical health issues, was examined in a cross-sectional study of children. The study found that FKBP5 promoter methylation in oral and blood cells was lower in abused children, suggesting that early physical abuse in childhood may impact epigenetic regulation of the stress response system (Everson et al., 2023). However, some studies indicate that FKBP5 DNA methylation levels do not mediate the relationship between CM and depression (Bustamante et al., 2018). Flasbeck and Brüne (2021) confirmed that FKBP5 methylation, which regulates GR sensitivity, is associated with psychopathology but not with the severity of childhood adversity, suggesting that the regulatory role of the FKBP5 gene in the interplay between ELS and depression remains ambiguous and requires further investigation in larger samples. Neuropeptide oxytocin (OT) can attenuate stress responses, and OT pathway dysregulation is observed in individuals with major depressive disorder (MDD). Therefore, the gene encoding oxytocin (OXT) might be a target for studying depression development. Studies found that OXT methylation levels in MDD inpatients were significantly lower than in healthy controls, suggesting that changes in the epigenetic characteristics of the OXT system may constitute a vulnerability factor for susceptibility to depression (Sanwald and Widenhorn-Müller, 2020).

#### 4.2.3 Histone modification

Despite having lower research intensity compared to DNA methylation, histone modifications have also been studied in depression, and the regulation of histone modifications holds promise as a new target for depression treatment. Histones are essential components of chromosomes, and common modifications include methylation, acetylation, ubiquitination, phosphorylation, and deamination (Grunstein, 1997). Histone modifications play a fundamental role in regulating gene expression, as they can influence chromatin relaxation or condensation by altering the affinity between histones and DNA double strands, thereby regulating gene transcription. To date, histone modifications have been investigated in depression, primarily focusing on histone acetylation and methylation, although their research intensity is lower compared to DNA methylation. Histone acetylation is controlled by histone acetyltransferases (HATs) and histone deacetylases (HDACs), responsible for adding and removing acetyl groups from histone tails, respectively. Animal experiments demonstrated that after exposure to social defeat stress, rats showing high locomotor activity and sustained exploratory behavior exhibited significantly increased levels of histone 3 at lysine 14 (H3K14) acetylation compared to rats displaying low activity and loss of exploration interest (Hollis et al., 2011). Another study on postmortem prefrontal cortex (PFC) histone modifications in MDD patients reported a positive correlation between trimethylation of histone 3 at lysine 4 (H3K4) and synaptic protein variant 1 (SYN1) (Cruceanu et al., 2013). These studies suggest that brain histone modifications may occur in response to severe stress events, leading to transcriptional changes and depression development. Thus, targeting histone modifications may offer a novel strategy to elucidate the pathogenesis of depression and a new direction for its treatment. Recently, Huang et al. found that acetate supplementation (a major source of acetyl coenzyme A) significantly improved depression-like behaviors in mice, substantially reduced transcript levels of HDAC2, HDAC5, HDAC7, and HDAC8, increased transcript levels of HAT and P300, and enhanced nuclear acetyl coenzyme A content, promoting acetylation levels of histones H3 and H4 (Huang et al., 2021), suggesting that acetate might exert antidepressant-like effects by enhancing acetylation of certain histones. Additionally, the potential application of histone deacetylase (HDAC) inhibitors in depression treatment has attracted research interest. A substantial body of preclinical studies, including rodents and studies by Nestler, have confirmed the promising antidepressant effects of HDAC inhibitors, indicating their potential for treating treatment-resistant depression (Fuchikami et al., 2016). Overall, there are currently no clinical trials evaluating the role of HDAC inhibitors in depression treatment. The potential of this therapy in treating depression patients remains to be developed. Furthermore, few studies have observed differences in histone acetylation and methylation levels between depression patients and normal controls, and investigations have not been conducted on a genome-wide scale. The occurrence and regulation of histone modifications in depression and how they change and modulate require further in-depth research.

#### 4.2.4 Non-coding RNA

Non-coding RNA (ncRNA) refers to RNA molecules transcribed from the genome that do not encode proteins. It mainly includes MicroRNA, Long non-coding RNA, and Circular RNA, serving as important regulatory factors within organisms. Their abnormal expression may participate in the occurrence and progression of human diseases, including depression.

MicroRNA (miRNA) is a class of non-coding, single-stranded RNA molecules approximately 22 nucleotides in length encoded by endogenous genes. In recent years, it has been discovered that miRNA dysregulation plays a crucial role in the onset and development of psychiatric disorders (Ma and Zhang, 2022). Ho et al. (2023), using human serum samples, found that compared to the healthy control group, six miRNAs (miR-542-3p, miR-181b-3p, miR-190a-5p, miR-33a-3p, miR-3690, and miR-6895-3p) were significantly downregulated in the serum of patients with depression. Lopez et al. (2014), through supplementary studies using human brain samples, cell assays, and clinical trial samples from depression patients, indicated differential expression of miR-1202 in the brains of individuals with depression. This implies a connection between miR-1202 and the pathophysiology of depression, making it a potential therapeutic target for depression. Therefore, miRNA may play a role in the onset and progression of depression by influencing gene dysregulation in peripheral blood or the brain, a phenomenon also observed in synaptic plasticity changes. Reduced brain plasticity, synaptic loss, and impaired synaptic genesis may lead to or exacerbate depression (Duman et al., 2016). Changes in synaptic function due to synaptic plasticity disorders are closely associated with alterations in miRNA expression. Research suggests that compared to healthy control subjects, major depressive disorder (MDD) patients exhibit significant differences in eight miRNAs (miR-215-5p, miR-192-5p, miR-202-5p, miR-19b-3p, miR-423-5p, miR-219a-2-3p, miR-511-5p, miR-483-5p) in the synaptic fraction of the dorsolateral prefrontal cortex (dlPFC) (Yoshino et al., 2021). These studies highlight the potential of miRNAs as biomarkers for diagnosing depression and as targets for antidepressant treatment. However, further research is needed to explore the regulation of miRNAs in different tissues, various physiological processes, and their involvement in the pathogenesis of depression.

Long non-coding RNA (lncRNA) was initially considered as “noise” in the gene transcription process, merely a byproduct of RNA polymerase II transcription without biological function. However, recent years of research have continuously demonstrated the involvement of lncRNA in various crucial biological processes, including chromatin silencing, epigenetic regulation, and chromatin modification (Hao et al., 2022). With the development of sequencing technologies, the relationship between lncRNA and depression has gradually emerged. Human clinical data studies have confirmed the association between lncRNA and depression, suggesting that lncRNA may serve as a potential biomarker for the diagnosis and treatment of depression (Cui et al., 2016; Shi et al., 2021a). In 2014, Liu et al. found the upregulation of four lncRNAs in the peripheral blood of patients with depression. Furthermore, through the construction of a co-expression network of differentially expressed lncRNA and mRNA, they identified three lncRNAs as potentially important regulators of mRNA expression, providing the first direct evidence supporting the molecular pathogenesis of lncRNA in regulating depression (Liu et al., 2014). Additionally, several lncRNAs have been discovered in the postmortem brain tissues of patients with depression. A Scottish cohort study based on a large family and population (*N* = 19,896, 2,659 cases, 17,237 controls) confirmed the specific expression of lncRNA RP1-269 M15.3 in the frontal cortex and nucleus accumbens of the brain tissues of patients with (MDD) (Zeng et al., 2017). Interestingly, Zhou et al. conducted RNA sequencing on the prefrontal cortex of 26 suicide victims with depression and 24 matched controls, identifying 23 differentially expressed lncRNAs. They also found that RP1-269 M15.3 was one of the most differentially expressed lncRNAs (Zhou et al., 2018). Furthermore, the expression of lncRNA RP1-269 M15.3 was replicated in the depression dataset released by the UK-Ireland Psychiatric Genomics Consortium, indicating the potential of this gene for the diagnosis and treatment of depression (Zeng et al., 2017). Therefore, the expression and associated genetic variations of lncRNA RP1-269 M15.3 may lead to changes in the depressive phenotype, influencing the onset and progression of depression. Further in-depth research is necessary.

Circular RNA (CircRNA) is a type of closed-loop molecule generated by reverse splicing of precursor mRNA, lacking a 5′ cap and a 3′ poly(A) tail. As an emerging field, recent research has indicated that circRNA participates in many biological functions, providing preliminary evidence for its role in regulating the pathological progression of depression (Li Z. et al., 2020; Gan et al., 2021). RNA sequencing of human peripheral blood has observed hundreds of circRNAs with expression levels significantly higher than their corresponding linear mRNAs, suggesting that circRNA can serve as biomolecules in standard clinical blood samples (Memczak et al., 2015). In another study on whole blood samples, researchers found that circFKBP8 was significantly reduced, and circMBNL1 expression was significantly increased in patients with depression, suggesting that whole blood circFKBP8 and circMBNL1 may be potential biomarkers and targets for antidepressant treatment, respectively (Shi et al., 2021b). More importantly, the molecular mechanisms of some circRNA molecules regulating depression have been elucidated in animal models. Huang et al. found that circSTAG1 was significantly decreased in the peripheral blood of patients with depression and in the hippocampal tissue and peripheral blood of chronic unpredictable mild stress (CUMS) mice. Further research demonstrated that this circRNA induced dysfunction of astrocytes and subsequent depression-like behavior by regulating the m6A methylation and degradation of fatty acid amide hydrolase (FAAH) mRNA (Huang et al., 2020). Additionally, researchers targeted circDYM to the brains of CUMS mice and found that circDYM significantly inhibited microglial activation, blood-brain barrier leakage, and peripheral immune cell infiltration, attenuating CUMS-induced astrocytic dysfunction and depression-like behavior (Yu et al., 2022). In conclusion, the importance of circRNA as potential biomarkers for depression diagnosis and as targets for antidepressant treatment is evident. However, independent validation and comprehensive functional studies are crucial for assessing the clinical value of these circRNA molecules.

#### 4.2.5 Epigenetic mechanism of antidepressants

Current psychotropic medications have been shown to influence epigenetic regulation, particularly by altering DNA methylation levels. Tricyclic antidepressants (TCAs) such as amitriptyline and imipramine, as well as selective serotonin reuptake inhibitors (SSRIs) like paroxetine, have been demonstrated to reduce promoter DNA methylation in primary rat astrocytes by inhibiting the activity of DNA methyltransferase 1. Additionally, antiepileptic drugs like carbamazepine and valproic acid can induce DNA demethylation in the prefrontal cortex and striatum (Dong et al., 2008; Zimmermann et al., 2012). Mood stabilizers and antiepileptic drugs, such as sodium butyrate, can trigger replication-independent active demethylation, as observed in human embryonic kidney cells (Detich et al., 2003). Research has identified methyl-CpG-binding protein 2 (MeCP2) as a transcriptional repressor that can influence DNA methylation, chromatin remodeling, and transcriptional inhibition processes. MeCP2 regulates processes like neuronal development, synaptogenesis, and maturation, thereby modulating BDNF expression (Bowen et al., 2004). Thus, MeCP2 could be a potential therapeutic target for new antidepressants, warranting further investigation of its role in depression.

Medications can also exert their effects by targeting histone deacetylases (HDACs) to influence epigenetic regulation. Sodium butyrate, an effective HDAC inhibitor, can improve cognitive impairment in depression patients, possibly by upregulating thyroxine receptor (Ttr), downregulating serotonin 2A receptor (Htr2a), and upregulating neurotrophic factors (Yamawaki et al., 2012; Valvassori et al., 2014). Combining the antidepressant fluoxetine with an HDAC inhibitor significantly enhances treatment efficacy by increasing acetylation of histone H4 and enriching RNA polymerase II on the brain-derived neurotrophic factor (Bdnf) gene promoter 3, leading to increased BDNF transcription (Schmauss, 2015). Fluoxetine combined with zinc reverses changes in hippocampal HDAC levels in depressed animals, suggesting a link between fluoxetine + Zn and HDAC regulation, with specific structural effects (Misztak et al., 2021). Furthermore, metabolic byproducts like lactic acid produced during physical activity and tricyclic antidepressant amitriptyline have been shown to have antidepressant effects by inhibiting HDAC activity (Mao et al., 2011; Karnib et al., 2019). However, non-specific HDAC inhibitors have limitations in terms of efficacy and safety, necessitating the development of more selective compounds. Three novel specific type I HDAC inhibitors (IN01, IN04, and IN14) were reported to have significantly higher cognitive-promoting and antidepressant effects than the tricyclic antidepressant desipramine, with IN14 exhibiting lower toxicity, indicating the potential of IN14 as a promising low-toxicity antidepressant (Martínez-Pacheco et al., 2020). Moreover, Reddy et al. constructed a single molecule composed of the key pharmacophores of HDAC inhibitors vorinostat and tubastatin-A, yielding compound 3-11, which displayed significant pan-HDAC inhibitory activity and potential to increase acetylation of histone H3 and microtubule-associated protein acetylation levels. Compound 5 exhibited better selectivity for class II HDACs, suggesting potential as a drug for treating depression and related psychiatric disorders (Reddy et al., 2019).

Antidepressants can also impact miRNAs, and upregulation of BDNF gene expression may be a key factor in the efficacy of antidepressants. The antidepressant paroxetine rapidly increases BDNF mRNA and protein expression, while the expression of miR-30a-5p, a post-transcriptional inhibitor of BDNF synthesis, is also increased (Mellios et al., 2008). This suggests that increased miR-30a-5p expression may inhibit BDNF protein expression. Furthermore, BDNF plays a crucial role in regulating synaptic structural remodeling and function. Researchers have shown that ginsenoside Rb1, via the miR-134-mediated BDNF signaling pathway, regulates hippocampal synaptic plasticity to improve depression symptoms (Wang et al., 2022). The NLRP3-mediated inflammasome is regulated by miRNA-27a, a key factor in depression development. Liquiritigenin, a flavonoid, can significantly suppress NLRP3-mediated inflammasome activation by upregulating miRNA-27a expression, exhibiting potent antidepressant properties (Li et al., 2021). Prolonged fluoxetine treatment in mice increases the level of miR-16 in the dorsal raphe nucleus, leading to reduced serotonin transporter (SERT) expression (Baudry et al., 2010). Baudry et al. demonstrated that miR-16 can inhibit SERT, suggesting that miR-16 may contribute to the therapeutic effects of SSRIs on monoaminergic neurons (Baudry et al., 2010).

### 4.3 Epigenetics of depression in adolescents

Adolescence is a critical developmental period marked by physical maturation, shifts in social roles, and the formation of self-regulatory mechanisms in the brain. During this time, the risk of mental health issues significantly increases, especially among adolescents, where the severity of psychological disorders, including major depressive disorder, bipolar disorder, anxiety disorders, and panic disorders, is particularly pronounced (Auerbach et al., 2018). Therefore, from an epigenetic perspective, this article aims to explore the research progress of depression in the adolescent population. In the present bibliometric analysis, we identified a total of 70 publications related to epigenetic research on adolescent depression, accounting for ~2% of the total. This number is relatively limited.

In a nested case-control study involving Chinese university students (cases, *n* = 50; controls, *n* = 100), NR3C1-16 CpG 10 methylation was found to significantly mediate the correlation between academic stress and anxiety symptoms, suggesting that NR3C1-16 CpG 10 DNA methylation could be a potential mechanism underlying adolescent anxiety (Hua et al., 2023). Similarly, a study involving Japanese medical students found the role of epigenetic regulatory factors in academic stress. Katsuura et al. assessed anxiety status and naturalistic stressor-responsive miRNAs in ten healthy medical students before, during, and after a national academic promotion exam, revealing that anxiety levels peaked on the day before the exam, accompanied by significant elevation of whole blood miR-144/144^*^ and miR-16 levels, indicating that miR-144/144^*^ and miR-16 might be part of the integrated response to natural stressors in university students (Katsuura et al., 2012). Changes in DNA methylation of the FK506 binding protein 5 (FKBP5) gene can modulate stress response, which is closely related to depression. Most relevant studies have focused on adults (Li M. et al., 2020). However, recently fewer studies have considered the association between FKBP5 DNA methylation and adolescent depression. Li et al. observed reduced FKBP5 methylation levels in adolescents with persistent depressive symptoms, although this reduction was not significant after multiple testing corrections (Li et al., 2022). In a nested case-control experiment, researchers found a link between childhood emotional abuse and increased risk of anxiety symptoms, but there was no significant correlation between childhood abuse and FKBP5 DNA methylation (Lai et al., 2022). In a clinical study by Piechaczek et al. (2019) involving cases of major depression (*n* = 148) and a normal control group (*n* = 143), participants carrying at least one copy of the FKBP5 CATT haplotype or at least one minor allele of various FKBP5 SNPs had the highest risk of being in the major depression group. This provides evidence for the interaction between FKBP5 gene variation and environmental stressors in adolescent depression. However, the role of FKBP5 methylation in adolescent depression remains ambiguous, necessitating further research into the regulatory mechanism of FKBP5 in adolescent depression and its interaction with the environment.

In summary, current research on the epigenetics of adolescent depression is still in its early stages and requires further exploration. If possible, researchers or clinical experts can utilize new techniques such as genome-wide association studies (GWAS) and whole-genome sequencing (WGS) to analyze genetic differences between the adolescent depression population and healthy individuals on a larger scale. By identifying critical gene targets and potential biomarkers, this approach can help us comprehend the complex mechanisms involved, reduce the occurrence of student depression, and timely detect, prevent, and improve their mental health.

### 4.4 Highlights and limitations

The highlights of this study lies in being the first bibliometric analysis of the field of depression epigenetics research over the past two decades. We employed various informative knowledge maps and tables to illustrate the developmental trajectory of this field, identify key leaders, uncover the scientific collaboration networks among authors and countries, elucidate the disciplinary foundation of this field, and analyze and discuss the research hotspots in the epigenetics of depression. The epigenetic research on adolescent depression, however, remains largely unexplored, presenting significant research potential. Therefore, this study offers insights into potential future directions and research methodologies for this area.

Nevertheless, there are several limitations to this study. Firstly, the publications were retrieved solely from the WoS database, which may limit the generalizability of the findings. Secondly, the analysis was restricted to research articles and reviews, excluding other relevant publications in the field such as book chapters and conference proceedings. Thirdly, only English-language literature indexed in the WoS database was collected, potentially leading to the exclusion of relevant non-English publications in the field, which might affect the overall summarization of the results. If feasible, a more comprehensive search strategy for prospective bibliometric analysis in this field should be designed to include a wider range of literature, unrestricted by language, document type, publication date, or database. Despite these limitations, we believe that the bibliometric approach used in this study provides novel insights into the development and current status of the field, as well as highlighting some challenges that hinder its progress.

## 5 Conclusion

In conclusion, the findings of this study unveil trends in the development of this field over the past two decades and hold promise for a better understanding of the scientific patterns between depression and epigenetics on a global scale. Firstly, researchers' interest in depression epigenetics has grown over the last 20 years, with the field's research momentum remaining steady in recent years. This suggests that the field is still in its growing phase and is expected to continue evolving in the future. Secondly, this research is predominantly interdisciplinary, involving the intersection of neuroscience and molecular science. For the future, we hope that researchers from diverse disciplines will engage in depression epigenetics research, working collaboratively to safeguard global mental health, particularly among adolescents, by reducing depression incidence and improving the lives of affected individuals. Thirdly, most research in this field is conducted by scholars from developed countries, often hindered by language, cultural background, and geographical barriers. Hence, breaking down these barriers and promoting global collaboration is imperative to advance research in this domain. Fourthly, over the past two decades, researchers have delved into various facets related to depression epigenetics and achieved significant progress. However, there is an insufficient focus on epigenetic studies of adolescent depression. Given the continuous advancements in epigenetic technology and the potential for large-scale longitudinal studies, the identification of potential biomarkers for adolescent depression remains an enticing possibility. Lastly, novel and comprehensive approaches are needed to advance our understanding of depression etiology and antidepressant mechanisms. Integrating epigenomic data with transcriptomics, proteomics, metabolomics, and other “omics” data will aid in comprehending the functional significance of observed inter-individual differences and the interactions between different biological systems influencing depression risk. In brief, our study provides guidance and reference for researchers to contemplate and delineate research directions, reducing efforts in exploring the boundaries of this field, and offering valuable information to further delve into this field.

## Data availability statement

The raw data supporting the conclusions of this article will be made available by the authors, without undue reservation.

## Author contributions

DY: Data curation, Formal analysis, Investigation, Methodology, Software, Validation, Visualization, Writing – original draft, Writing – review & editing. YM: Data curation, Methodology, Software, Visualization, Writing – original draft. ZA: Funding acquisition, Writing – review & editing. SZ: Funding acquisition, Writing – review & editing.
